# Single Autoantibody Positivity in Pediatric Type 1 Diabetes: Serological Profiles, Clinical Trajectories and Implications for Early Disease Monitoring—A Case Series of Seven Patients

**DOI:** 10.3390/life16091500

**Published:** 2026-09-08

**Authors:** Natasha Yaneva, Trifon T. Popov, Meri Petrova, Adelina Yordanova, Margarita Arshinkova, Dobroslav Kyurkchiev, Ekaterina Kurteva

**Affiliations:** 1 Clinic for Pediatric Endocrinology, Diabetes and Metabolic Diseases, University Children’s Hospital “Prof. Ivan Mitev”, 1606 Sofia, Bulgaria; 102268@students.mu-sofia.bg (T.T.P.); marshinkova@medfac.mu-sofia.bg (M.A.); 2 Department of Pediatrics, Medical Faculty, Medical University of Sofia, 1431 Sofia, Bulgaria; 3 University Hospital St. Ivan Rilski, Laboratory of Clinical Immunology, Department of Clinical Immunology, Medical Faculty, Medical University of Sofia, 1431 Sofia, Bulgaria; meri.petrova1234@gmail.com (M.P.); adelina.d.yordanova@gmail.com (A.Y.); dkyurkchiev@medfac.mu-sofia.bg (D.K.); e.kurteva@medfac.mu-sofia.bg (E.K.)

**Keywords:** type 1 diabetes, autoantibodies, single autoantibody positivity, apparent seroreversion, autoantibody dynamics, pediatric screening, lifestyle counseling, case series

## Abstract

Background: Type 1 diabetes (T1D) incidence is rising at 3–5% per year. Autoantibody (AAB) screening identifies at-risk children pre-symptomatically, yet the clinical significance of single AAB positivity and its short-term trajectory remain incompletely characterized. Methods: Five T1D-associated AABs (anti-GAD65, anti-IA2, anti-ZnT8, ICA, IAA) were measured in 210 Bulgarian children (160 with first-degree T1D relatives, 50 controls). Seven single-AAB-positive children received lifestyle counseling (low-glycemic-index diet, physical activity ≥ 60 min/day) and were reassessed at 3 or 6 months with repeat AAB panels, HbA_1c_, blood glucose and C-peptide. Results: Anti-GAD65 was the most frequent single AAB (*n* = 3), followed by anti-ZnT8 (*n* = 2), anti-IA2 (*n* = 1) and IAA (*n* = 1). None developed a second AAB or metabolic abnormalities. Titer dynamics were heterogeneous: two children showed apparent seroreversion (in one, substantially confounded by concurrent immunosuppressive therapy for an unrelated condition), one showed a ~15-fold titer reduction while remaining seropositive, one showed a minimal titer decrease, and three displayed mildly increasing titers. None progressed to multiple autoantibody positivity, and all maintained normal metabolic profiles. Conclusions: Favorable short-term outcomes were observed during follow-up of children with single autoantibody positivity. These findings suggest that single AAB positivity may follow a non-progressive course in some children. However, this study design cannot distinguish the contribution of lifestyle counseling from natural variability. Prospective controlled studies with larger cohorts, HLA stratification and longer follow-up are needed to determine whether lifestyle interventions can independently alter autoantibody dynamics and delay T1D progression.

## 1. Introduction

Type 1 diabetes (T1D) is the most common form of diabetes in children and adolescents, with a global incidence rising at 3–5% per annum and over 1.1 million affected children worldwide [1,2,3,4]. Unlike type 2 diabetes, T1D has a distinct autoimmune pathogenesis driven predominantly by CD8+ T cell-mediated destruction of pancreatic β-cells, with islet autoantibodies (AABs) serving as the earliest detectable serological markers of this process [5,6].

**Figure 1:**
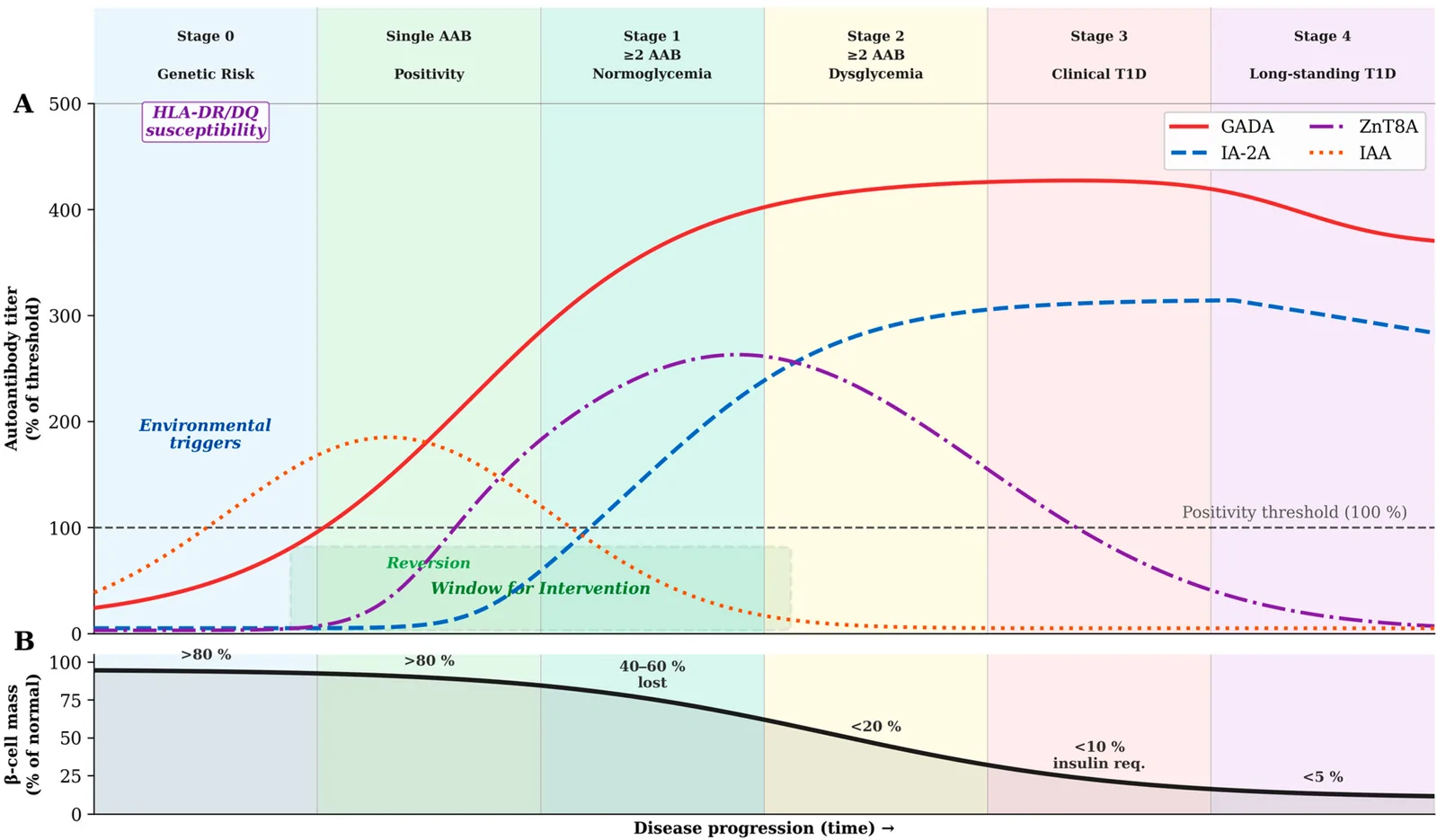
Stages of type 1 diabetes mellitus (T1D) and schematic representation of functional pancreatic β-cell mass decline over time. (**A**) Autoantibody titer dynamics (% of positivity threshold) across disease stages. Stage 0 represents the period before multiple autoantibody (AAB) formation, during which genetic susceptibility (HLA-DR/DQ), environmental and infectious factors trigger the immune response. The “Single AAB Positivity” stage precedes Stage 1 and denotes isolated seroconversion, with the green-bordered area indicating the potential window for reversion and intervention. Stage 1 is asymptomatic with normoglycemia and ≥2 AABs. Stage 2 is asymptomatic with ≥2 AABs and dysglycemia. Stage 3 is characterized by fulfillment of diagnostic criteria for diabetes, presence of AABs and clinical symptoms. Stage 4 is long-standing T1D. The horizontal dashed line indicates the positivity threshold (100%). Colored curves represent individual autoantibody trajectories: GADA (red solid line), IA-2A (blue dashed line), ZnT8A (purple dash-dot line) and IAA (orange dotted line); (**B**) Progressive decline of functional pancreatic β-cell mass (% of normal) over the course of disease progression. The black solid line represents the estimated residual β-cell mass at each stage, with annotated percentages indicating the approximate remaining functional capacity (>80% at Stages 0–1, 40–60% lost at Stage 2, <20% at Stage 3, <10% requiring insulin at clinical diagnosis, and <5% in long-standing T1D) The rightward arrow on the x-axis indicates the direction of disease progression over time. In childhood, T1D often presents with diabetic ketoacidosis (DKA)—a life-threatening acute complication. Population-based AAB screening programs—such as the Fr1da study in Germany (>200,000 children screened) [9,10]—have demonstrated that pre-symptomatic detection leads to lower HbA_1c_ at Stage 3 onset, higher C-peptide concentrations, and substantially reduced DKA frequency [11].

Specific AABs detectable in T1D include anti-glutamic acid decarboxylase 65 (anti-GAD65), anti-insulinoma-associated antigen 2 (anti-IA2), anti-zinc transporter 8 (anti-ZnT8), islet cell autoantibodies (ICA) and insulin autoantibodies (IAA). Based on autoantibody status and glycemic profile, T1D is classified into four stages: Stage 1 (multiple AABs, normoglycemia, asymptomatic), Stage 2 (multiple AABs, dysglycemia, asymptomatic), Stage 3 (clinical diabetes with AABs) and Stage 4 (long-standing T1D) [7,8]. This staging framework reflects that T1D is a chronic autoimmune process beginning years before clinical onset, which allows pre-symptomatic detection and early intervention (Figure 1). Single autoantibody positivity does not fulfill the immunological criteria for Stage 1, which requires two or more islet autoantibodies [7,8]. Nevertheless, single-AAB-positive children represent an at-risk population warranting longitudinal surveillance.

**Figure 2:**
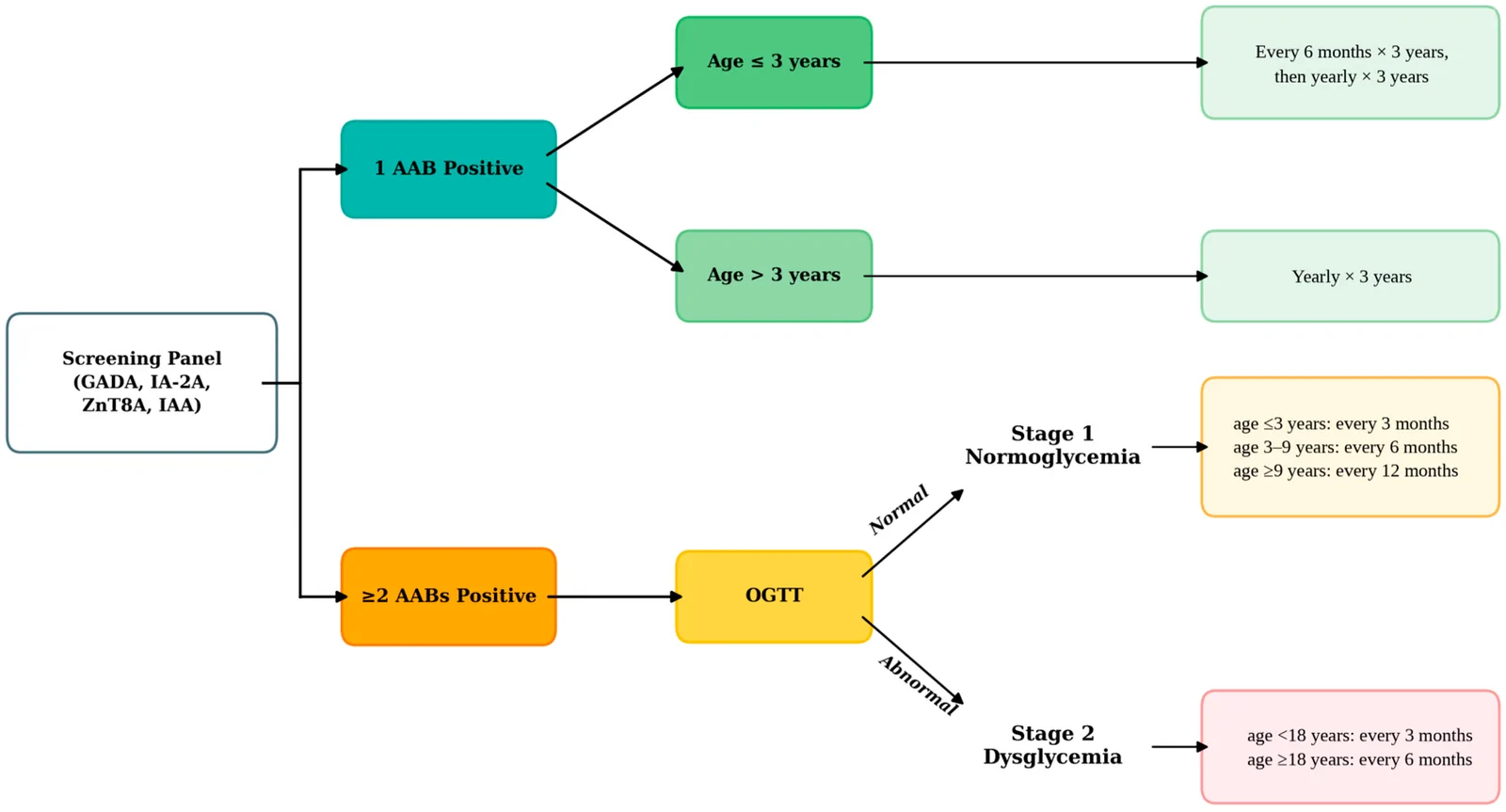
Recommendations for clinical management of single or multiple AAB positivity during T1D screening according to ISPAD. For single AAB positivity, monitoring includes AAB levels, glycated hemoglobin (HbA_1c_) and random blood glucose. For multiple AABs, monitoring includes HbA_1c_, random blood glucose and, if possible, continuous glucose monitoring.

According to ISPAD guidelines [7,8], single-AAB-positive children under 3 years should be monitored every 6 months (for 3 years, then annually for 3 more years), while children over 3 years are monitored annually for 3 years. Monitoring includes AAB levels, HbA_1c_ and random blood glucose; multiple AABs require an oral glucose tolerance test (OGTT) and more frequent follow-up (Figure 2).

The 10-year cumulative risk for T1D with a single AAB is 14.5% (95% CI 10.3–18.7%), rising to 69.7% (95% CI 65.1–74.3%) with multiple AABs [12]. However, single-AAB positivity is not a uniform risk category: TrialNet data show that single IA-2A positivity carries a 5-year Stage 3 risk of 50.9%, versus 12.0% for GADA and 9.6% for IAA [13]—differences that matter for clinical risk assessment.

Reversion from single AAB positivity to seronegativity is a recognized phenomenon. In the TEDDY study, reversion occurred in 24% of children with single AAB positivity; 85% of these reversions occurred within 2 years of seroconversion. Children who reverted had a substantially lower post-reversion T1D risk (0.14 per 100 person-years) compared with those who remained persistently single AAB positive (1.8 per 100 person-years) [14].

Despite growing evidence on T1D staging and population-based screening, limited data are available on single-AAB-positive children. First, the natural history of single autoantibody positivity is not well defined, with limited prospective data on factors determining whether positivity is transient or persistent. Second, although the accelerator hypothesis [15] suggests a role for lifestyle factors in early-stage autoimmunity, prospective data on their impact on autoantibody dynamics in single-AAB-positive children are lacking. Third, real-world data from Eastern European populations, where T1D screening programs are still in their early stages, are virtually absent from the international literature.

In Bulgaria, between April 2024 and February 2026, selective screening for T1D-associated AABs was conducted in 210 children: 160 with first-degree relatives with T1D and 50 controls without family history. Seven children with single AAB positivity were identified [16]. This case series aims to characterize the serological profiles and short-term clinical trajectories of these seven children following early detection and structured lifestyle counseling, and to describe autoantibody dynamics during short-term follow-up in this understudied population.

## 2. Materials and Methods

We conducted this study at the Clinic for Pediatric Endocrinology, Diabetes and Metabolic Diseases, University Children’s Hospital “Prof. Ivan Mitev,” Sofia, Bulgaria. Between April 2024 and February 2026, we screened 210 children for T1D-associated autoantibodies: 160 with at least one first-degree relative with T1D and 50 controls without a family history. Seven children (4.4% of the high-risk group) tested positive for a single islet autoantibody and comprise the present case series. No additional selection criteria were applied; these seven patients represent all single-AAB-positive children identified during the screening period.

The design and population-level results of this screening program have been reported previously [16]; that publication presented only cross-sectional autoantibody prevalence data. The present study provides an independent longitudinal analysis of individual patient trajectories, serial autoantibody measurements, metabolic follow-up, and lifestyle intervention outcomes, none of which were included in the original report.

Venous blood samples were collected by standard phlebotomy into serum separator tubes. After centrifugation, serum was aliquoted to prevent repeated freeze–thaw cycles and stored at −80 °C until analysis. All five T1D-associated autoantibodies (anti-GAD65, anti-IA2, anti-ZnT8, ICA, IAA) were measured simultaneously in the Laboratory of Clinical Immunology at University Hospital “St. Ivan Rilski,” Sofia, using chemiluminescent immunoassay (CLIA) on the MAGLUMI X3 platform (Snibe Diagnostics, Shenzhen, China). The inter-assay coefficients of variation (CV) were less than 15% for all analytes throughout the study period. The manufacturer-recommended positivity thresholds were: anti-GAD65 > 17 IU/mL, anti-IA2 > 28 IU/mL, IAA > 20 IU/mL, ICA > 28 U/mL, and anti-ZnT8 > 10 AU/mL.

Positive autoantibody results were determined from a single sample. We did not repeat sampling or confirm results with alternative assays, which we acknowledge as a limitation of this study. Both baseline and follow-up measurements were performed on the same MAGLUMI X3 instrument (SNIBE Co., Ltd., Shenzhen, China) using identical assay kits and, where possible, the same reagent lots to ensure analytical comparability between time points.

Apparent seroreversion was defined as a decline from above the positivity cut-off at baseline to below that threshold at follow-up, confirmed on the same analytical platform under identical assay conditions. We recognize that a single follow-up measurement does not confirm durable reversion; we use the term descriptively.

### 2.1. Case Presentations

All seven patients received structured lifestyle counseling at the time of initial result disclosure. Patients N1 and N4 were reassessed at 3 months; all others at 6 months. Patient N1 (age 1.0 year) was seen earlier because of a strong family history and the clinical significance of early-onset autoantibody positivity. Patient N4 was reassessed at 3 months due to intercurrent autoimmune encephalitis and Drug Reaction with Eosinophilia and Systemic Symptoms (DRESS) syndrome, necessitating immunosuppressive therapy that could independently alter AAB dynamics. The remaining five patients were reassessed at 6 months in accordance with the study protocol, which adopted this follow-up interval to maximize data collection within the available funding period. This 6-month interval is more frequent than the annual reassessment schedule recommended by ISPAD for single-AAB-positive children over 3 years of age [7,8], providing additional data points within the study period.

Dietary recommendations included strict avoidance of granulated sugar, white bread, and other high-glycemic-index foods, with emphasis on complex carbohydrates, vegetables and fiber-rich foods. Regular physical activity of at least 60 min per day was also encouraged. These recommendations were communicated verbally to parents and reinforced during follow-up visits. Adherence was monitored through parental self-report, although no validated assessment instrument was employed. Supplementary Table S1 provides individual details on metabolic parameters and follow-up outcomes.

Among the seven children with a single positive autoantibody (AAB), three were male (42.9%), and four were female (57.1%), with ages ranging from 1.0 to 15.3 years. All participants had a first-degree relative with T1D. Individual clinical and serological profiles are presented in Table 1 and described below.

Case no. 1: Female, aged 1.0 year, BMI Z-score +1.79 (overweight). First-degree relative with T1D (sister); mother with Hashimoto’s thyroiditis. No autoimmune comorbidities. Single positive AAB: anti-GAD65 (29.50 IU/mL).

Case no. 2: Female, aged 5 years 11 months, BMI Z-score −0.1 (normal weight). First-degree relative with T1D (father). No autoimmune comorbidities identified. Single positive AAB: anti-ZnT8 (14.8 AU/mL).

Case no. 3: Male, aged 4 years 10 months, BMI Z-score −0.7 (normal weight). First-degree relative with T1D (brother). No known allergies or autoimmune conditions. Single positive AAB: anti-IA2 (56.90 U/mL).

Case no. 4: Female, aged 13 years 2 months, BMI Z-score +1.3 (overweight). First-degree relative with T1D (mother). Significant autoimmune comorbidity: autoimmune encephalitis, treated with intravenous immunoglobulin (IVIG, 2 g/kg for 5 days) and pulse methylprednisolone (1 g daily for 5 days). Between baseline and follow-up screening, developed DRESS syndrome, necessitating additional pulse methylprednisolone (500 mg i.v. daily for 3 days), intravenous corticosteroid (1 mg/kg daily) and IVIG (2 g/kg). Single positive AAB: anti-GAD65 (153.00 IU/mL).

Case no. 5: Male, aged 9 years, BMI Z-score +1.0 (upper-normal). First-degree relative with T1D (sister); mother with Hashimoto’s thyroiditis. No celiac disease or thyroid autoimmunity. Single positive AAB: anti-GAD65 (124.00 IU/mL).

Case no. 6: Female, aged 15 years 4 months, BMI Z-score +1.7 (overweight). Mother with T1D. No autoimmune comorbidities. Single positive AAB: IAA (49.60 IU/mL).

Case no. 7: Male, aged 6 years 1 month, BMI Z-score −2.9 (underweight). First-degree relative with T1D (father); mother with Hashimoto’s thyroiditis. Normal thyroid function; celiac disease excluded. Single positive AAB: anti-ZnT8 (347.0 AU/mL).

Serological results are presented in Table 2. The most common positive AAB was anti-GAD65, detected in three of the seven patients (42.9%). Anti-ZnT8 was the second most frequent, present in two children (28.6%). Anti-IA2 and IAA were positive in patients N3 and N6, respectively. ICA were negative in all patients.

### 2.2. Follow-Up Assessments and Clinical Outcomes

Follow-up assessments comprised measurements of autoantibody levels, glycated hemoglobin, blood glucose, C-peptide, and body mass index (BMI). Individual metabolic and anthropometric follow-up data are provided in Supplementary Table S1. Serological follow-up data are presented in Table 3.

No patients developed a second positive AAB during follow-up, and all maintained normal HbA_1c_, blood glucose and C-peptide levels, indicating no metabolic progression. A decline below the positivity cutoff was observed in one patient (N1; anti-GAD65: 29.50 to 2.18 IU/mL). Patient N4 also showed a titer decline below the cutoff (anti-GAD65: 153.00 to 13.00 IU/mL); however, this outcome was confounded by concurrent immunosuppressive therapy with high-dose immunoglobulin and corticosteroids for autoimmune encephalitis and DRESS syndrome, and therefore cannot be attributed to lifestyle modification.

**Figure 3:**
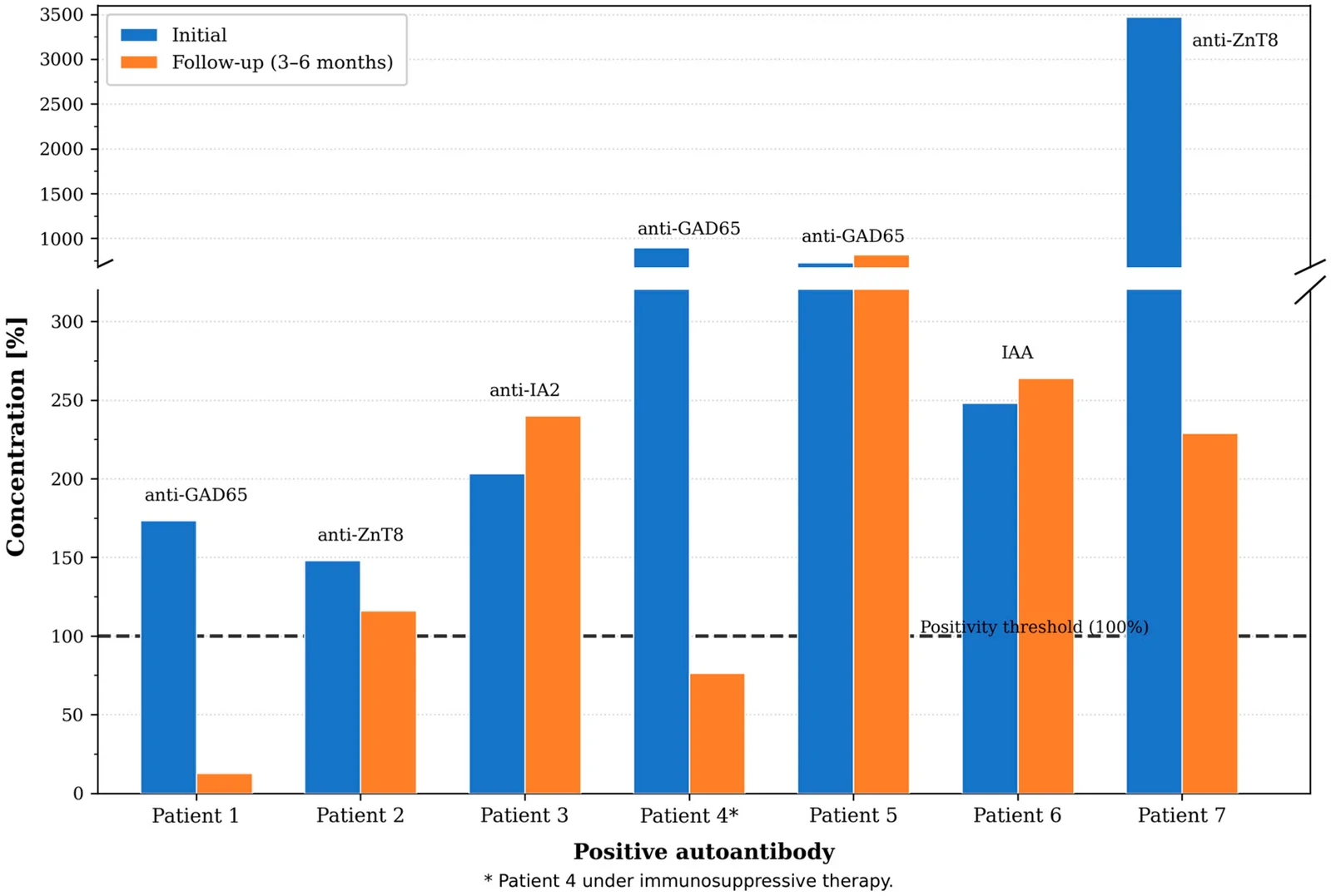
Relative concentrations of the positive pancreatic islet autoantibody in each patient at baseline (blue) and at follow-up (orange). Patients 1 and 4 were reassessed at 3 months, whereas all other patients—at 6 months. Autoantibody concentrations were normalized to assay-specific positivity cut-offs (anti-GAD65 ≥ 17.00 IU/mL; anti-IA2 ≥ 28.00 U/mL; IAA ≥ 20.00 IU/mL; anti-ZnT8 ≥ 10.00 AU/mL) and expressed as a percentage of the respective threshold. The horizontal dashed line indicates the 100% clinical cutoff; values above it indicate positive AAB levels.

Among anti-ZnT8-positive patients, N7 showed a 15-fold reduction in antibody levels (347.00 to 22.90 AU/mL). Nevertheless, the follow-up value remained above the positivity threshold (>10.00 AU/mL), indicating persistent seropositivity despite the quantitative decline. Patient N2 showed only a minimal decrease. Three patients showed mildly increasing titers while maintaining normal metabolic profiles: N3 (anti-IA2: 56.90 to 67.20 U/mL), N5 (anti-GAD65: 124.00 to 139.00 IU/mL), and N6 (IAA: 49.60 to 52.80 IU/mL). The intra-assay coefficient of variation for the MAGLUMI platform is less than 10%, and the observed changes in these three patients (+18%, +12%, and +6%, respectively) are near or within this range, suggesting that these minor fluctuations may reflect analytical variability rather than true immunological change (Figure 3).

None of the children with rising titers developed a second AAB or showed glycemic deterioration during the short observation period. However, a follow-up duration of 3 to 6 months is insufficient to determine whether these fluctuations predict eventual progression. Extended surveillance over 2 to 3 years is required before drawing definitive conclusions about prognostic significance.

## 3. Discussion

### 3.1. Serological Characterization and Risk Stratification

Anti-GAD65 was the most frequent single autoantibody (*n* = 3), consistent with TEDDY data, which identify GADA as a dominant early marker of islet autoimmunity [17,18]. Anti-ZnT8 was the second most prevalent (*n* = 2), found exclusively in pre-pubertal children. ZnT8A seroconversion occurs later than other islet autoantibodies—at a median of approximately 3–4 years in BABYDIAB [19] and 4–5 years in TEDDY [17], generally appearing after initial seroconversion to IAA, GADA or IA-2A. Our two ZnT8A-positive patients (N2, age 5.9; N7, age 6.1) are close to but slightly above this expected window, consistent with age-dependent expression patterns reported by Couper et al. [20]. Both exhibited low-to-moderate titers (N2: 14.80 AU/mL; N7: 347.00 AU/mL), which reflects the known variability in ZnT8A levels during early seroconversion. The ZnT8A assay used in this study (MAGLUMI CLIA) has shown excellent concordance with ELISA (Cohen’s κ = 0.94, 97.1% overall agreement) in an independent method comparison [21]. Confirmatory electrochemiluminescence (ECL) testing substantially improves the positive predictive value for islet autoantibody detection [22,23]. Our observation of divergent ZnT8A dynamics (N7: 15-fold titer reduction; N2: minimal decrease) aligns with Wenzlau et al.’s demonstration of marked inter-individual heterogeneity in ZnT8A decline kinetics (half-lives: 26–530 weeks) [24].

### 3.2. Contextual Interpretation of Autoantibody Dynamics

The observed titer trajectories in our cohort (Section 2.2), including apparent seroreversions and declines, are consistent with TEDDY data, which indicate that 24% of children positive for a single autoantibody revert to seronegativity, primarily within two years, and subsequently exhibit a markedly reduced risk of T1D (0.14 vs. 1.8 per 100 person-years) [14]. Autoantibody transience, defined as a single positive result followed by persistent negativity [25], is well established in early childhood. In the DIPP study, 49.3% of IAA-positive children reverted to negativity, with inverse seroconversion associated with a prolonged delay to diagnosis [18]. Similarly, Colman et al. reported transient islet autoimmunity in 14% of at-risk infants [26]. Longitudinal modeling demonstrates that titer trajectory has prognostic significance [27]. In a large cohort study, children with a single autoantibody that later reverted to negative had the lowest risk of progression, with a 5-year type 1 diabetes risk of only 1.6% [28]. However, baseline autoantibody positivity in the present cohort was determined from a single measurement on one analytical platform without orthogonal assay confirmation. Some uncertainty therefore remains regarding the interpretation of apparent seroreversion events, and confirmatory testing by an independent methodology would strengthen future analyses.

The divergent anti-GAD65 trajectories observed in patient N4 (153.00 to 13.00 IU/mL, influenced by immunosuppressive therapy) and N5 (124.00 to 139.00 IU/mL, persistently seropositive) exemplify the heterogeneity of single autoantibody dynamics. Elevated GADA titers predict progression to multiple autoantibodies [29], supporting the need for ongoing surveillance of patient N5.

### 3.3. Lifestyle Modification: Rationale and Evidence

The theoretical basis for this intervention rests upon the accelerator hypothesis, which posits that insulin resistance acts as an independent accelerator of β-cell destruction in genetically susceptible individuals, whereby excess adiposity amplifies β-cell stress, antigen shedding and autoimmune activation [15,30]. Several cohort studies support the prediction that heavier children develop T1D earlier [31,32,33], and Meah et al. reported modestly increased diabetes risk with higher BMI percentile among 1310 single-AAB-positive TrialNet participants [34].

Baseline BMI Z-score data from our cohort span a wide range (Table 1). Three of seven patients (N1: +1.79, N4: +1.30, N6: +1.70) had BMI Z-scores in the overweight range, while N5 (+1.0) was at the upper limit of normal, and N2 (−0.1), N3 (−0.7), and N7 (−2.9) were normal or underweight. The presence of single-AAB positivity across the full BMI spectrum is consistent with genetic risk being the primary driver of islet autoimmunity, regardless of body weight [15,30], while metabolic stress may contribute as a cofactor in some but not all cases, supporting an individualized approach to lifestyle recommendations.

Neither insulin resistance nor direct immunological parameters were measured in this cohort. The accelerator hypothesis is presented here solely as biological background to contextualize the lifestyle recommendations; it does not represent a demonstrated mechanism in our study.

A low-glycemic-index diet was recommended to reduce postprandial glucose excursions and β-cell secretory burden, based on evidence that such diets improve insulin sensitivity in obese children (homeostatic model assessment for insulin resistance (HOMA-IR) reduction, *p* = 0.007) [35]. Prospective data from the ABIS birth cohort (*n* = 16,415) indicate that high physical activity in early childhood reduces T1D incidence by approximately 50–58% [36], and additional studies confirm that regular moderate-to-vigorous physical activity improves insulin sensitivity in youth with or at risk for T1D [37,38,39,40]. However, no randomized trial has yet tested whether dietary or activity interventions can alter autoantibody titers in genetically at-risk children.

When individual autoantibody trajectories are compared against baseline BMI, no consistent pattern emerges. Among overweight patients, N1 showed a complete decline below the positivity cutoff (29.50 → 2.18 IU/mL), and N4 also declined below the cutoff (confounded by immunosuppressive therapy), whereas N6 (IAA: 49.60 → 52.80 IU/mL) showed a modest rise despite elevated BMI (+1.70). This suggests that adiposity alone does not determine serological outcomes. Patient N7 (BMI Z-score −2.9), the leanest in the cohort, showed the largest absolute titer reduction (347.00 → 22.90 AU/mL), further supporting that BMI does not linearly predict serological outcome.

These data provide a biological rationale for potential benefits of dietary and activity changes but serve as theoretical background only. The absence of a control group, the small sample size and the lack of objective adherence measures prevent causal conclusions. Whether the autoantibody changes observed in this cohort reflect a lifestyle effect or natural fluctuation remains undetermined.

### 3.4. Implications for Longitudinal Monitoring of Single-Autoantibody-Positive Children

In our series, outcomes ranged from apparent seroreversion (N1, N4), through substantial titer reduction (N7) and minimal decrease (N2), to persistent or mildly rising titers (N3, N5, N6). Five of seven patients remained seropositive at follow-up. A single measurement is insufficient for risk stratification. Prospective monitoring allows earlier recognition and milder presentation at diagnosis [11].

ISPAD 2024 recommends continued periodic reassessment of single-AAB-positive children [8]. Our data support this approach. Residual T1D risk persists even after apparent seroreversion [14], and optimal monitoring intervals remain undefined.

### 3.5. Emerging Disease-Modifying Therapies and C-Peptide Preservation

Teplizumab recently received approval for delaying Stage 2 to Stage 3 progression in individuals with multiple autoantibodies [41]. Our single-AAB-positive patients are not currently eligible. Nonetheless, early identification through structured monitoring preserves the option for timely intervention should they progress to Stage 2.

### 3.6. Immunosuppressive Therapy and Autoantibody Decline: The Case of Patient N4

Patient N4 received high-dose immunoglobulin and corticosteroid therapy for autoimmune encephalitis and DRESS syndrome. The observed anti-GAD65 decline (153.00 → 13.00 IU/mL) cannot be attributed solely to lifestyle modification, which complicates interpretation of AAB dynamics in the setting of immunomodulatory treatment.

### 3.7. Strengths and Limitations

This study is among the few detailed case-level reports of single-AAB-positive children from Eastern Europe. Strengths include a complete five-autoantibody panel at baseline and follow-up, longitudinal paired measurements that enabling trajectory assessment, and detailed clinical phenotyping. The study examines the natural history of single-AAB positivity identified through targeted familial screening.

Several limitations should be acknowledged. First, the small sample size (*n* = 7) of this descriptive case series precludes statistical inference and generalizability. Second, the follow-up period was limited to 3–6 months; extended surveillance is necessary to assess the durability of AAB reversion and to identify late progressors. This constraint is attributable to the funding timeline, which ended at the 6-month post-screening point. Third, the lack of a control group prevents causal attribution. The short follow-up period and the absence of a control group preclude conclusions about the independent effect of lifestyle modification. In addition, patient N4’s concurrent immunosuppressive therapy constitutes a significant confounding variable. Adherence to lifestyle recommendations was evaluated solely through parental self-report without a validated instrument, restricting the ability to quantify its independent effect or to distinguish it from natural autoantibody fluctuation. The absence of HLA typing impedes genetic risk stratification. Finally, reliance on a single analytical platform (MAGLUMI) without confirmatory testing by an alternative methodology (such as ELISA or ECL) limits interpretability.

## 4. Conclusions

Seven Bulgarian children with single autoantibody positivity identified through familial screening were monitored over six months. Autoantibody trajectories were heterogeneous: two children showed apparent seroreversion, one (N7) had a substantial (~15-fold) titer reduction while remaining above the positivity threshold, one (N2) showed a minimal decrease, and three (N3, N5, N6) had persistent or mildly increasing titers. In one case (N4), the titer decline was strongly confounded by concurrent high-dose corticosteroid and immunoglobulin therapy for autoimmune encephalitis and DRESS syndrome. None of the children progressed to multiple autoantibody positivity. Anti-GAD65 was the predominant autoantibody. These results confirm that a single serological assessment is insufficient for risk stratification; periodic reassessment is recommended for all children with single autoantibody positivity. Familial screening combined with lifestyle counseling is a feasible approach for early immune surveillance in settings with limited access to disease-modifying therapies. However, the short follow-up period and the absence of a control group preclude conclusions about the independent effect of lifestyle modification, and prospective controlled studies with larger cohorts are needed to address this question.

## Figures and Tables

**Table 1:** Demographic and clinical characteristics of the seven patients.

Patient	Age (Years)	Gender	BMI Z-Score	Family History of T1D	Autoimmune Comorbidities
N1	1.0	Female	+1.79	Yes (sister)	No
N2	5.9	Female	−0.1	Yes (father)	No
N3	4.8	Male	−0.7	Yes (brother)	No
N4	13.2	Female	+1.3	Yes (mother)	Yes (autoimmune encephalitis)
N5	9.0	Male	+1.0	Yes (sister)	No
N6	15.3	Female	+1.7	Yes (mother)	No
N7	6.1	Male	−2.9	Yes (father)	No

**Table 2:** Initial serological results of the seven patients with a single positive AAB. Positive values (exceeding the assay-specific positivity cutoff) are shown in bold.

Patient	Anti-GAD65 [pos. > 17.00 IU/mL]	Anti-IA2 [pos. > 28.00 IU/mL]	IAA [pos. > 20.00 IU/mL]	ICA [pos. > 28.00 U/mL]	Anti-ZnT8 [pos. > 10.00 AU/mL]
N1	**29.50 IU/mL**	2.00 U/mL	4.04 IU/mL	6.69 U/mL	1.00 AU/mL
N2	0.68 IU/mL	2.00 U/mL	9.61 IU/mL	3.26 U/mL	**14.8 AU/mL**
N3	3.69 IU/mL	**56.90 U/mL**	4.54 IU/mL	14.1 U/mL	1.00 AU/mL
N4	**153.00 IU/mL**	2.00 U/mL	8.40 IU/mL	19.80 U/mL	1.00 AU/mL
N5	**124.00 IU/mL**	2.00 U/mL	8.38 IU/mL	12.40 U/mL	1.00 AU/mL
N6	3.67 IU/mL	2.00 U/mL	**49.60 IU/mL**	3.73 U/mL	1.00 AU/mL
N7	2.98 IU/mL	2.00 U/mL	8.61 IU/mL	5.34 U/mL	**347.0 AU/mL**

**Table 3:** Follow-up serological data of the patients (3 or 6 months after the initial screening). Positive values are shown in bold. Arrows indicate direction of change relative to baseline. In the Outcome column, bold text highlights clinically favorable serological changes, including a decline below the positivity cutoff or a substantial decrease in autoantibody levels.

Patient	Anti-GAD65 [pos. > 17.00 IU/mL]	Anti-IA2 [pos. > 28.00 IU/mL]	IAA [pos. > 20.00 IU/mL]	ICA [pos. > 28.00 U/mL]	Anti-ZnT8 [pos. > 10.00 AU/mL]	Outcome	Follow-Up
N1	2.18↓	2.00	4.04	6.69	1.00	**Below cutoff**	3 months
N2	2.23	2.00	4.28	11.52	**11.6↓**	**Minimal** **decrease**	6 months
N3	2.43	**67.2↑**	4.86	20.00	1.00	Persistent	6 months
N4 *	13.00↓	2.00	4.36	6.84	1.00	**Below cutoff**	3 months
N5	**139.00↑**	2.00	7.78	11.60	1.00	Persistent	6 months
N6	2.27	2.00	**52.80↑**	4.52	1.00	Persistent	6 months
N7	2.46	2.00	7.41	4.25	**22.90↓**	**Decreased (15×), still positive**	6 months

* N4: concurrent immunosuppressive therapy (see Section 3.6).

## Data Availability

The original contributions presented in the study are included in the article; further inquiries can be directed to the corresponding author.

## Supplementary Materials

The following supporting information can be downloaded at: https://www.mdpi.com/article/10.3390/life16091500/s1, Table S1: Individual metabolic, anthropometric and glycemic follow-up data for seropositive children (*n* = 7).

## Abbreviations

The following abbreviations are used in this manuscript:

AABs	autoantibodies
anti-GAD65 (GADA)	anti-glutamic acid decarboxylase 65
anti-IA2 (IA-2A)	anti-insulinoma-associated antigen 2
anti-ZnT8 (ZnT8A)	anti-zinc transporter 8
BMI	body mass index
CI	confidence interval
CLIA	chemiluminescent immunoassay
CV	coefficient of variation
DKA	diabetic ketoacidosis
DRESS	drug reaction with eosinophilia and systemic symptoms
ECL	electrochemiluminescence
ELISA	enzyme-linked immunosorbent assay
HbA1c	glycated hemoglobin
HLA	human leukocyte antigen
HOMA-IR	homeostatic model assessment for insulin resistance
IAA	insulin autoantibodies
ICA	islet cell autoantibodies
ISPAD	International Society for Pediatric and Adolescent Diabetes
IVIG	intravenous immunoglobulin
OGTT	oral glucose tolerance test
T1D	type 1 diabetes

## References

[1] Buzzetti R., Zampetti S., Pozzilli P. (2020). Impact of obesity on the increasing incidence of type 1 diabetes. Diabetes Obes. Metab..

[2] Dahlquist G. (2006). Can we slow the rising incidence of childhood-onset autoimmune diabetes? The overload hypothesis. Diabetologia.

[3] Knip M., Reunanen A., Virtanen S.M., Nuutinen M., Viikari J., Åkerblom H.K. (2008). Does the secular increase in body mass in children contribute to the increasing incidence of type 1 diabetes?. Pediatr. Diabetes.

[4] International Diabetes Federation (2021). IDF Diabetes Atlas.

[5] American Diabetes Association (2013). Diagnosis and classification of diabetes mellitus. Diabetes Care.

[6] Atkinson M.A., Eisenbarth G.S., Michels A.W. (2014). Type 1 diabetes. Lancet.

[7] Besser R.E.J., Bell K.J., Couper J.J., Ziegler A., Wherrett D.K., Knip M., Speake C., Casteels K., Driscoll K.A., Jacobsen L. (2022). ISPAD clinical practice consensus guidelines 2022: Stages of type 1 diabetes in children and adolescents. Pediatr. Diabetes.

[8] Haller M.J., Bell K.J., Besser R.E.J., Casteels K., Couper J.J., Craig M.E., Elding Larsson H., Jacobsen L., Lange K., Oron T. (2024). ISPAD clinical practice consensus guidelines 2024: Screening, staging, and strategies to preserve beta-cell function in children and adolescents with type 1 diabetes. Horm. Res. Paediatr..

[9] Schill S., Friedl N., Winkler C., Haupt F., Scholz M., Stock J., Bonifacio E., Ziegler A.G., Achenbach P. (2025). Screening for islet autoantibodies in childhood: Insights from the Fr1da study on early-stage type 1 diabetes prevalence and progression. Diabetol. Stoffwechs..

[10] Bonifacio E., Winkler C., Achenbach P., Ziegler A.G. (2024). Effect of population-wide screening for presymptomatic early-stage type 1 diabetes on paediatric clinical care. Lancet Diabetes Endocrinol..

[11] Hummel S., Carl J., Friedl N., Winkler C., Kick K., Stock J., Reinmüller F., Ramminger C., Schmidt J., Lwowsky D. (2023). Children diagnosed with presymptomatic type 1 diabetes through public health screening have milder diabetes at clinical manifestation. Diabetologia.

[12] Ziegler A.G., Rewers M., Simell O., Simell T., Lempainen J., Steck A., Winkler C., Ilonen J., Veijola R., Knip M. (2013). Seroconversion to multiple islet autoantibodies and risk of progression to diabetes in children. JAMA.

[13] Sims E.K., Cuthbertson D.D., Bosi E., Evans-Molina C., Redondo M.J., Nathan B.M., Ismail H.M., Jacobsen L.M., Sosenko J. (2023). Single islet antigen 2 antibody-positive (IA2A Ab+) children exhibit significant metabolic abnormalities and 5-year type 1 diabetes risk. Diabetes.

[14] Vehik K., Lynch K.F., Schatz D.A., Akolkar B., Hagopian W., Rewers M., She J.X., Simell O., Toppari J., Ziegler A.G. (2016). Reversion of β-cell autoimmunity changes risk of type 1 diabetes: TEDDY study. Diabetes Care.

[15] Wilkin T.J. (2009). The accelerator hypothesis: A review of the evidence for insulin resistance as the basis for type I as well as type II diabetes. Int. J. Obes..

[16] Yaneva N., Petrova M., Yordanova A., Popov T., Arshinkova M., Kyurkchiev D., Kurteva E. (2026). The first selective screening for type 1 diabetes in a pediatric population in Bulgaria. J. Clin. Med..

[17] Vehik K., Bonifacio E., Lernmark Å., Yu L., Williams A., Schatz D.A., Rewers M., She J.X., Hagopian W., Toppari J. (2020). Hierarchical order of distinct autoantibody spreading and progression to type 1 diabetes in the TEDDY study. Diabetes Care.

[18] Pöllänen P.M., Ryhänen S.J., Toppari J., Ilonen J., Vähäsalo P., Veijola R., Siljander H., Knip M. (2020). Dynamics of Islet Autoantibodies During Prospective Follow-Up From Birth to Age 15 Years. J. Clin. Endocrinol. Metab..

[19] Achenbach P., Lampasona V., Landherr U., Koczwara K., Krause S., Grallert H., Winkler C., Pflüger M., Illig T., Bonifacio E. (2009). Autoantibodies to zinc transporter 8 and SLC30A8 genotype stratify type 1 diabetes risk. Diabetologia.

[20] Couper J.J., Oakey H., Penno M.A.S., Wentworth J.M., Watson K., Brown J.D., Huynh D., Thomson R.L., Craig M.E., Davis E.A. (2026). Evolution of islet autoantibodies in the Environmental Determinants of Islet Autoimmunity (ENDIA) prospective cohort. Diabetologia.

[21] Danese E., Piona C., Rizza M., Tiziani E., Pighi L., Morotti E., Salvagno G.L., Mattiuzzi C., Maffeis C., Lippi G. (2025). A Comparative Evaluation of the Chemiluminescence Immunoassay and ELISA for the Detection of Islet Autoantibodies in Type 1 Diabetes. Diagnostics.

[22] Miao D., Guyer K.M., Dong F., Jiang L., Steck A.K., Rewers M., Eisenbarth G.S., Yu L. (2013). GAD65 autoantibodies detected by electrochemiluminescence assay identify high risk for type 1 diabetes. Diabetes.

[23] Jia X., He L., Miao D., Waugh K., Rasmussen C.G., Dong F., Steck A.K., Rewers M., Yu L. (2021). High-affinity ZnT8 autoantibodies by electrochemiluminescence assay improve risk prediction for type 1 diabetes. J. Clin. Endocrinol. Metab..

[24] Wenzlau J.M., Walter M., Gardner T.J., Frisch L.M., Yu L., Eisenbarth G.S., Ziegler A.-G., Davidson H.W., Hutton J.C. (2010). Kinetics of the Post-Onset Decline in Zinc Transporter 8 Autoantibodies in Type 1 Diabetic Human Subjects. J. Clin. Endocrinol. Metab..

[25] Kukko M., Kimpimäki T., Korhonen S., Kupila A., Simell S., Veijola R., Simell T., Ilonen J., Simell O., Knip M. (2005). Dynamics of diabetes-associated autoantibodies in young children with human leukocyte antigen-conferred risk of type 1 diabetes recruited from the general population. J. Clin. Endocrinol. Metab..

[26] Colman P.G., Steele C., Couper J.J., Beresford S.J., Powell T., Kewming K., Pollard A., Gellert S., Tait B., Honeyman M. (2000). Islet autoimmunity in infants with a type I diabetic relative is common but is frequently restricted to one autoantibody. Diabetologia.

[27] Korneva K.G., Chichevatov D.A., Strongin L.G., Zagainov V.E. (2025). Role of longitudinal measurement of autoantibodies in predicting type 1 diabetes mellitus in children. Russ. Med..

[28] Ghalwash M., Anand V., Ng K., Dunne J.L., Lou O., Lundgren M., Hagopian W.A., Rewers M., Ziegler A.G., Veijola R. (2024). Data-driven phenotyping of presymptomatic type 1 diabetes using longitudinal autoantibody profiles. Diabetes Care.

[29] You L., Salami F., Tamura R., Törn C., Vehik K., Hagopian W.A., Rewers M.J., McIndoe R.A., Toppari J., Ziegler A.G. (2025). Predictors of Transitions From GADA as the Initial Autoantibody to Multiple Autoantibodies of Type 1 Diabetes in Children at Risk by a Dynamic Prediction Model. Pediatr. Diabetes.

[30] Wilkin T.J. (2012). The convergence of type 1 and type 2 diabetes in childhood: The accelerator hypothesis. Pediatr. Diabetes.

[31] Kibirige M., Metcalf B.S., Renuka R., Wilkin T.J. (2003). Testing the accelerator hypothesis: The relationship between body mass and age at diagnosis of type 1 diabetes. Diabetes Care.

[32] Knerr I., Wolf J., Reinehr T., Stachow R., Grabert M., Schober E., Rascher W., Holl R.W. (2005). The ‘accelerator hypothesis’: Relationship between weight, height, body mass index and age at diagnosis in a large cohort of 9,248 German and Austrian children with type 1 diabetes mellitus. Diabetologia.

[33] Dabelea D., D’Agostino R.B., Mayer-Davis E.J., Pettitt D.J., Imperatore G., Dolan L.M., Pihoker C., Hillier T.A., Marcovina S.M., Linder B. (2006). Testing the accelerator hypothesis: Body size, β-cell function, and age at onset of type 1 (autoimmune) diabetes. Diabetes Care.

[34] Meah F.A., DiMeglio L.A., Greenbaum C.J., Blum J.S., Sosenko J.M., Pugliese A., Geyer S., Xu P., Evans-Molina C. (2016). The relationship between BMI and insulin resistance and progression from single to multiple autoantibody positivity and type 1 diabetes among TrialNet Pathway to Prevention participants. Diabetologia.

[35] Visuthranukul C., Sirimongkol P., Prachansuwan A., Pruksananonda C., Chomtho S. (2015). Low-glycemic index diet may improve insulin sensitivity in obese children. Pediatr. Res..

[36] Ludvigsson J., Sohail T. (2025). Early Life Physical Activity May Reduce the Risk of Developing Type 1 Diabetes: The Longitudinal ABIS Study. Diabetes Metab. Res. Rev..

[37] Ungethüm K., Jolink M., Hippich M., Lachmann L., Haupt F., Winkler C., Hummel S., Pitchika A., Kordonouri O., Ziegler A.-G. (2019). Physical activity is associated with lower insulin and C-peptide during glucose challenge in children and adolescents with family background of diabetes. Diabet. Med..

[38] Aljawarneh Y.M., Wardell D.W., Wood G.L., Rozmus C.L. (2019). A systematic review of physical activity and exercise on physiological and biochemical outcomes in children and adolescents with type 1 diabetes. J. Nurs. Scholarsh..

[39] Szadkowska A. (2021). Exercise and sport—Challenges and benefits for the children and adolescents with type 1 diabetes. Pediatr. Endocrinol. Diabetes Metab..

[40] Landt K.W., Campaigne B.N., James F.W., Sperling M.A. (1985). Effects of exercise training on insulin sensitivity in adolescents with type I diabetes. Diabetes Care.

[41] Herold K.C., Bundy B.N., Long S.A., Bluestone J.A., DiMeglio L.A., Dufort M.J., Gitelman S.E., Gottlieb P.A., Krischer J.P., Linsley P.S. (2019). An anti-CD3 antibody, teplizumab, in relatives at risk for type 1 diabetes. N. Engl. J. Med..

