# Supplementary material for: Single Autoantibody Positivity in Pediatric Type 1 Diabetes: Serological Profiles, Clinical Trajectories and Implications for Early Disease Monitoring—A Case Series of Seven Patients
Source: Life (Basel). 2026 Sep 8;16(9):1500. doi: 10.3390/life16091500 (PMC13608845; doi:10.3390/life16091500)
Supplement: Supplementary file 1 [file life-16-01500-s001.zip › life-4502684-supplementary.pdf]

Supplementary Table S1. Individual metabolic, anthropometric and glycemic follow-up data for seropositive children ($n = 7$).

Patient	Positive Autoantibody	Baseline BMI Z-score	Follow-up BMI Z-score	ΔBMI Z-score	HbA_{1c} (%)	Fasting Glucose (mmol/L)	C-peptide (ng/mL)[a]	Dietary Adherence[b]	Metabolic Outcome
N1	anti-GAD65	+1.79	+2.13	+0.34	5.14	4.75	1.33	Full	Normal glucose; elevated BMI
N2	anti-ZnT8	−0.10	+0.36	+0.46	4.33	4.40	2.30	Full	Normal
N3	anti-IA2	−0.70	−0.80	−0.10	4.60	5.10	0.56	Full	Normal
N4[*]	anti-GAD65	+1.30	+0.42	−0.88	5.20	5.43	1.62	Full	Normal
N5	anti-GAD65	+1.00	+0.77	−0.23	4.53	4.76	1.15	Full	Normal
N6	IAA	+1.70	+1.10	−0.60	4.60	5.11	2.12	Partial	Normal; overweight
N7	anti-ZnT8	−2.90	−0.54	+2.36	4.99	4.51	0.36	Full	Normal

[a] C-peptide reference range: 0.35–4.48 ng/mL. [b] Dietary adherence assessed by parental self-report. [*] Patient N4 was under immunosuppressive therapy at the time of follow-up. Abbreviations: BMI, body mass index; Δ, change from baseline to follow-up; HbA_{1c}, glycated hemoglobin. All children received lifestyle counseling comprising low-glycemic index diet and physical activity ≥60 min/day. BMI Z-scores calculated using WHO Child Growth Standards (0–5 years) and WHO Growth Reference (5–19 years).
